# Pharmacological inactivation of CHK1 and WEE1 induces mitotic catastrophe in nasopharyngeal carcinoma cells

**DOI:** 10.18632/oncotarget.4020

**Published:** 2015-05-21

**Authors:** Joyce P.Y. Mak, Wing Yu Man, Jeremy P.H. Chow, Hoi Tang Ma, Randy Y.C. Poon

**Affiliations:** ^1^ Division of Life Science, Center for Cancer Research, and State Key Laboratory of Molecular Neuroscience, Hong Kong University of Science and Technology, Clear Water Bay, Hong Kong

**Keywords:** DNA damage checkpoint, mitosis, mitotic catastrophe, nasopharyngeal carcinoma, WEE1

## Abstract

Nasopharyngeal carcinoma (NPC) is a rare but highly invasive cancer. As radiotherapy is the primary treatment for NPC, this offers a rationale to investigate if uncoupling the DNA damage responses can sensitize this cancer type. The G_2_ DNA damage checkpoint is controlled by a cascade of protein kinases: ATM/ATR, which phosphorylates CHK1/CHK2, which in turn phosphorylates WEE1. A number of small molecule inhibitors have been developed against these kinases as potential therapeutic agents. Here we demonstrated that compare to that in immortalized nasopharyngeal epithelial cells, ATR, CHK1, and WEE1 were overexpressed in NPC cell lines. Inhibitors of these kinases were unable to promote extensive mitotic catastrophe in ionizing radiation-treated NPC cells, indicating that they are not very effective radiosensitizer for this cancer. In the absence of prior irradiation, however, mitotic catastrophe could be induced with inhibitors against CHK1 (AZD7762) or WEE1 (MK-1775). NPC cells were more sensitive to WEE1 inactivation than nasopharyngeal epithelial cells. Targeting CHK1 and WEE1 together induced more extensive mitotic catastrophe than the individual components alone. Taken together, our results show that NPC cells depend on CHK1 and WEE1 activity for growth and that inhibitors of these kinases may serve as potential therapeutics for NPC.

## INTRODUCTION

Nasopharyngeal carcinoma (NPC) is a highly invasive cancer with poor prognosis. Although NPC is relatively rare in most parts of the world, high incidence rates are found in southern China and Southeast Asia [1]. The standard treatment for NPC includes surgical resection and radiotherapy [2]. Treatment outcomes for advanced NPC have been poor due to metastasis and recurrence [3]. Although chemotherapeutic compounds are used in combination with radiotherapy to control advanced NPC, they are restricted to traditional agents such as cisplatin and 5-flurouracil. Effective radiosensitizers for NPC are still to be established.

After DNA damage, a surveillance mechanism termed the G_2_ DNA damage checkpoint prevents entry into mitosis. The checkpoint involves the activation of a kinase cascade initiating with ATM and the related ATR. Activated ATR/ATM phosphorylates residues in the SQ/TQ domain of CHK1 and CHK2, stimulating the activity of these effector kinases [4]. CHK1/CHK2 then acts on all three isoforms of the CDC25 family to suppress their activities [5]. CHK1 also phosphorylates and activates WEE1 in yeast [6, 7], and, in *Xenopus*, phosphorylates and activates WEE1 by promoting 14-3-3 binding [8, 9]. Inhibition of CDC25 or activation of WEE1 promotes Thr14/Tyr15 phosphorylation of CDK1, thereby preventing damaged cells from entering mitosis. Although there are considerable overlaps in the pathway, the prevailing view is that while the ATM-CHK2 pathway primarily responds to DNA double-strand breaks, the ATR-CHK1 pathway is activated by a broader spectrum of DNA abnormalities. Premature inactivation of the G_2_ DNA damage checkpoint can trigger a process often termed mitotic catastrophe, which is characterized by precocious mitosis followed by apoptosis or mitotic slippage [10].

Mounting evidence indicates that in addition to its role in checkpoints, the ATR-CHK1-WEE1 axis also plays an essential role in the unperturbed cell cycle. Deletion of ATR [11, 12], CHK1 [13], or WEE1 [14] results in embryonic lethality. Inhibition of these kinases during normal S phase facilitates activation of cyclin E-CDK2, which in turn leads to unscheduled initiation of DNA replication, thereby inducing DNA damage in a mechanism that is not yet fully understood [15].

One focus of the development of inhibitors of the checkpoint kinase cascade is for their use as chemosensitizers or radiosensitizers [16]. DNA damage is particular relevant for NPC for several reasons [17]. Firstly, radiotherapy remains the main treatment for NPC. Secondly, Epstein-Barr virus infection (a major etiological factor for NPC) induces DNA damage. Finally, the DNA damage checkpoint is frequently impaired in NPC. Nevertheless, the effects of targeting the DNA damage checkpoint kinases have not been studied in NPC. Only one study shows that treatment with a CHK1 inhibitor called Gö6976 sensitizes NPC cells to radiation and cisplatin [18]. Here we present evidence that the components of the kinase cascade are overexpressed in NPC in comparison to immortalized nasopharyngeal cells. Furthermore, NPC cell growth was inhibited by targeting CHK1 and WEE1.

## RESULTS

### Overexpression of the ATR-CHK1-WEE1 axis in nasopharyngeal carcinoma cell lines

**Figure 1:**
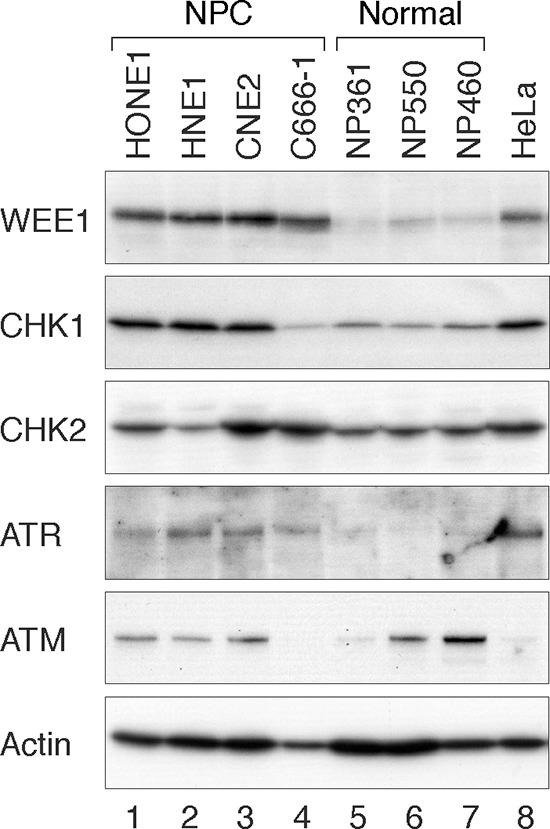
The ATR-CHK1-WEE1 axis is overexpressed in NPC cell lines Several NPC (HONE1, HNE1, CNE2, and C666-1) and immortalized nasopharyngeal (NP) epithelial cell lines (NP361, NP550, and NP460) were analyzed. Lysates from HeLa cells were also loaded for comparison. Cell-free extracts were prepared and the indicated proteins were detected by immunoblotting.

The G_2_ DNA damage checkpoint is frequently dysregulated in NPC [17]. To determine if components of the checkpoint kinase cascade are expressed in NPC cells, lysates from a number of NPC cell lines (C666-1, CNE2, HNE1, and HONE1) were prepared and analyzed with immunoblotting. Several telomerase-immortalized nasopharyngeal epithelial cell lines (NP361, NP460, and NP550) were used for comparison. The specificity of some of the antibodies used is shown in Figure S1. We found that WEE1 was upregulated in all the NPC cell lines examined (Figure 1). To a lesser extent, CHK1 and ATR were also upregulated in most NPC cell lines (except C666-1 for CHK1). No such correlation was observed for ATM and CHK2. These data indicated that ATR, CHK1, and WEE1 are in general upregulated in NPC cell lines. In this study, we mainly used HONE1 and HNE1 as representative NPC cell lines (both poorly differentiated [19]) and NP460 as nasopharyngeal epithelial cells.

### Targeting checkpoint kinases can disrupt the G_2_ DNA damage checkpoint in irradiated NPC cells

**Figure 2:**
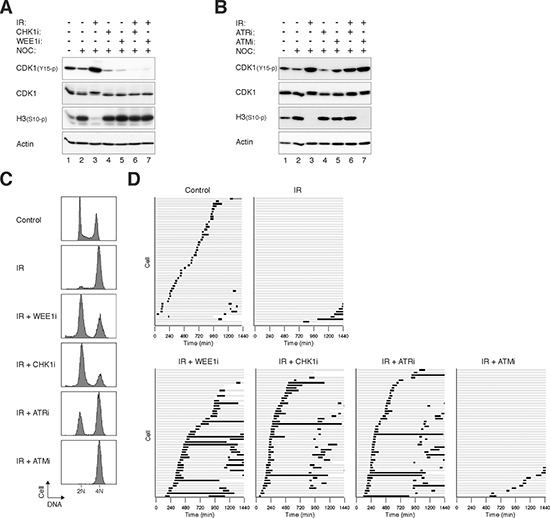
Targeting ATR, CHK1, and WEE1 abrogates the G_2_ DNA damage checkpoint in irradiated NPC cells **A.** Disruption of the G_2_ DNA damage checkpoint by inhibition of WEE1 and CHK1. HONE1 cells were either mock-treated or irradiated with 10 Gy of ionizing radiation (IR). After 16 h, the cells were incubated with buffer, 500 nM of MK-1775 (WEE1i), or 50 nM of AZD7762 (CHK1i). Nocodazole (NOC) was also applied to trap cells in mitosis. The cells were harvested after another 8 h. Lysates were prepared and the indicated proteins were detected with immunoblotting. Uniform loading of lysates was confirmed by immunoblotting for actin. **B.** ATRi but not ATMi abrogates the IR-mediated checkpoint. HONE1 cells were either untreated or irradiated with 10 Gy of IR. After 16 h, the cells were incubated with 2.5 μM of VE-821 (ATRi) or 5 μM of KU-60019 (ATMi). Nocodazole (NOC) was also applied to trap the cells in mitosis. After 8 h, the cells were harvested and analyzed with immunoblotting. Uniform loading of lysates was confirmed by immunoblotting for actin. **C.** Targeting the ATR-CHK1/CHK2-WEE1 axis overcomes IR-induced G_2_ arrest. HONE1 cells were either mock-treated or irradiated with 10 Gy of IR. After 16 h, the cells were incubated with buffer, WEE1i (500 nM), CHK1i (50 nM), ATRi (2.5 μM) or ATM (5 μM). After 8 h, the cells were harvested and analyzed with flow cytometry. **D.** Targeting the ATR-CHK1/CHK2-WEE1 axis induces mitosis in irradiated cells. HONE1 cells expressing histone H2B-mRFP were either mock-treated or irradiated with 10 Gy of IR. After 16 h, the cells were incubated with buffer, WEE1i (250 nM), CHK1i (250 nM), ATRi (5 μM) or ATM (5 μM). Individual cells were then tracked for 24 h with time-lapse microscopy. Each horizontal bar represents one cell (*n* = 50). Light grey: interphase; black: mitosis (from DNA condensation to anaphase); truncated bars: cell death. Note that only one of the daughter cells was tracked after mitosis.

Radiotherapy is the primary treatment for NPC [2]. We next examined if disrupting the radiation-induced G_2_ DNA damage checkpoint could increase cytotoxicity in NPC cells. The G_2_ DNA damage checkpoint in HONE1 was activated after treatment with ionizing radiation (IR), as indicated by an accumulation of CDK1^Tyr15^ phosphorylation and a decrease of histone H3^Ser10^ phosphorylation (Figure 2A, lane 3). Addition of an inhibitor of WEE1 (MK-1775 [20], designated WEE1i herein) abolished CDK1^Tyr15^ phosphorylation and restored histone H3^Ser10^ phosphorylation (lane 7), indicating that inhibition of WEE1 abrogated the checkpoint. Note that nocodazole was also included in these experiments to trap the checkpoint-abrogated cells in mitosis. Likewise, AZD7762 (CHK1i herein), an inhibitor of CHK1 (IC_50_: 5 nM) and CHK2 (IC_50_: < 10 nM) [21], was also able to disrupt the IR-induced G_2_ arrest (lane 6).

We also examined the effects of targeting upstream kinases of the checkpoint. Figure 2B shows that 2.5 μM of VE-821 (ATRi herein), a specific inhibitor of ATR [22], was able to overcome the checkpoint, reversing both the phosphorylation of CDK1^Tyr15^ and histone H3^Ser10^. However, the checkpoint was not disrupted by an ATM inhibitor (5 μM of KU-60019 [23] (ATMi herein)).

To verify that the G_2_ cell cycle arrest could be attenuated by checkpoint inhibitors, DNA contents were analyzed with flow cytometry (Figure 2C). IR induced mainly a G_2_/M arrest in HONE1 cells. Addition of WEE1i for another 8 h resulted in cells containing mainly G_1_ DNA contents, indicating that the damaged cells were forced into the cell cycle. Similar results were obtained using CHK1i and ATRi. In agreement with the above observations, ATMi was unable to overcome the G_2_ arrest under these conditions.

We further verified the fates of checkpoint-abrogated cells directly using live-cell imaging. After HONE1 cells were irradiated and arrested at G_2_ (16 h), they were challenged with checkpoint inhibitors before individual cells were tracked using time-lapse microscopy. In contrast to control cells, which entered and exited mitosis asynchronously, the majority of IR-treated cells stopped cell cycle progression and remained in interphase during the 24 h imaging period (Figure 2D). The arrested cells were able to enter mitosis after the checkpoint was abrogated with WEE1i, CHK1i, or ATRi (but not ATMi). Checkpoint abrogation resulted in mitosis that was in general longer than that during unperturbed cell cycle.

Similar results were obtained with another NPC cell line (HNE1) (Figure S2A), indicating that the effects of the checkpoint inhibitors were not limited to HONE1. As with HONE1 cells, HNE1 responded to IR-mediated damage by arresting at G_2_ phase (Figure S2B) with CDK1^Tyr15^ phosphorylation (Figure S2C). Inhibitors including WEE1i, CHK1i, and ATRi were able to abrogate the checkpoint in HNE1 cells.

Interestingly, the same concentration of WEE1i did not affect the G_2_ DNA damage checkpoint in nasopharyngeal epithelial cells (Figure S3). This is also consistent with the results that NP460 cells were less sensitive to WEE1i as a standalone compound than NPC cells (see later). These results suggest that nasopharyngeal epithelial cells and NPC cells have different susceptibility to WEE1i.

Although targeting components of the kinase cascade could abrogate the G_2_ DNA damage checkpoint in NPC cells, this did not result in significant cytotoxicity. This was supported by the absence of sub-G_1_ population (Figure 2C), cleaved PARP1 (data not shown), and apoptotic cells (Figure 2D). Similarly, no significant apoptosis was detected after checkpoint abrogation in HNE1 cells (Figure S2A). These results indicated that abrogation of the G_2_ DNA damage in NPC cells did not result in massive mitotic cell death as observed in other cell lines such as HeLa (Figure S4). Moreover, longer-term analysis (up to 6 days) indicated that WEE1i did not further reduce cell growth compare to cells treated with IR alone (Figure S5).

Collectively, these data indicate that pharmacological inhibition of the ATR-CHK1/CHK2-WEE1 pathway can attenuate IR-mediated arrest in NPC cells. However, this checkpoint abrogation does not promote mitotic catastrophe.

### NPC cells are more sensitive to inhibition of WEE1 than nasopharyngeal epithelial cells

**Figure 3:**
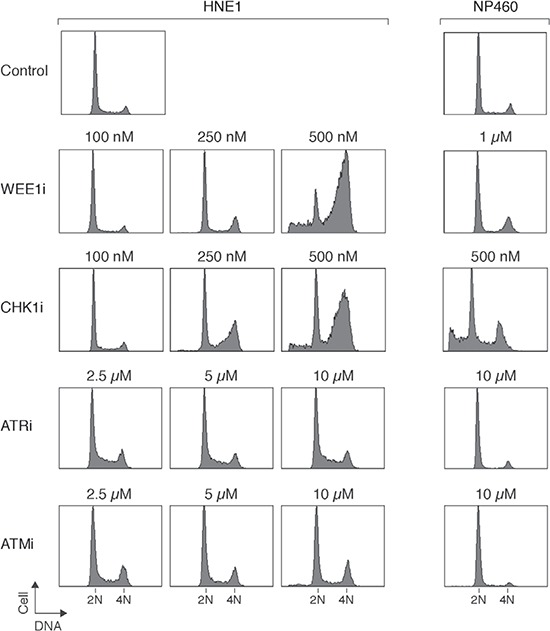
Inhibition of WEE1 specifically sensitizes NPC cells compare to nasopharyngeal epithelial cells HNE1 and nasopharyngeal epithelial cells (NP460) were exposed to the indicated concentrations of MK-1775 (WEE1i), AZD7762 (CHK1i), VE-821 (ATRi), or KU-60019 (ATMi). After 24 h (for HNE1) or 48 h (for NP460), the cells were harvested and analyzed with flow cytometry. NP460 cells were treated for longer because they have a slower doubling time than the NPC cells. The positions of 2N and 4N DNA contents are indicated.

Given that abolition of the IR-mediated checkpoint did not significantly enhance apoptosis in NPC cells, we next tested if targeting the checkpoint in the absence of DNA damage could be more effective in inducing cytotoxicity. The basis of this is that checkpoint inhibitors could mainly target cells during S phase (instead of mainly G_2_ cells after DNA damage). Figure 3 shows that incubation of HNE1 cells with 500 nM of WEE1i or CHK1i enriched cells in G_2_/M or the later part of S phase. In marked contrast, ATRi and ATMi did not induce similar cell cycle delay even when used at up to 10 μM. Similar sensitivity to WEE1i and CHK1i and lack of cell cycle effects of ATRi and ATMi were observed with another NPC cell line HONE1 (Figure S6A), excluding the possibility that the differential effect was specific for HNE1 only. As expected, WEE1i or CHK1i decreased cell proliferation in a dose-dependent manner (Figure S6B).

**Figure 4:**
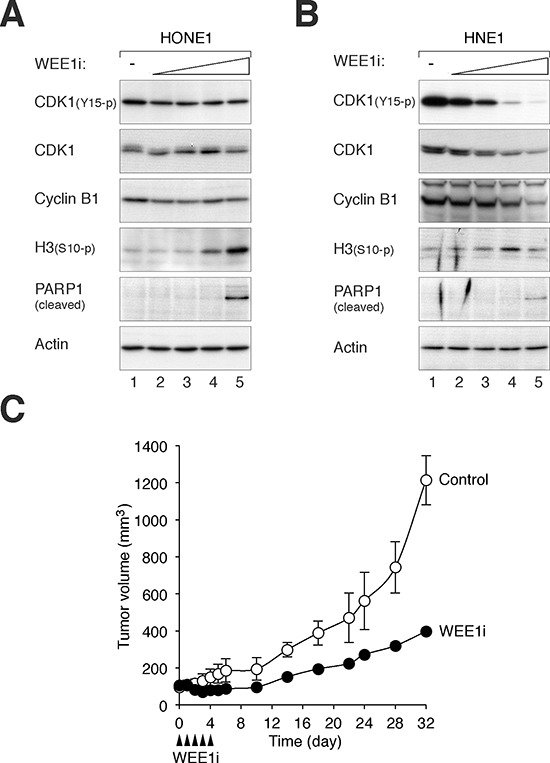
Inhibition of WEE1 induces mitotic catastrophe and inhibits cell growth **A.** WEE1i promotes mitotic catastrophe in HONE1 cells. HONE1 cells were incubated with either buffer or increasing concentrations of WEE1i (100 nM, 250 nM, 500 nM, and 1 μM) for 24 h. Lysates were prepared and the expression of the indicated proteins was detected with immunoblotting. Equal loading of lysates was confirmed by immunoblotting for actin. **B.** WEE1i promotes mitotic catastrophe in HNE1 cells. HNE1 cells were incubated with either buffer or increasing concentrations of WEE1i (100 nM, 250 nM, 500 nM, and 1 μM) for 24 h. Lysates were prepared and the expression of the indicated proteins was detected with immunoblotting. Equal loading of lysates was confirmed by immunoblotting for actin. **C.** WEE1i inhibits tumor growth in mouse xenografts. HONE1 cells were injected subcutaneously into nude mice. WEE1i (closed arrow head) was delivered at the indicated time points as described in Materials and Methods. The volume of the tumor was measured on different days (mean ± SD; *n* = 3).

Importantly, immortalized nasopharyngeal epithelial cells were less sensitive to WEE1i than NPC cells. While 0.5 – 1 μM of WEE1i was able to induce G_2_/M responses in NPC cells, NP460 was not affected (Figure 3). Both HONE1 (Figure 4A) and HNE1 (Figure 4B) responded to WEE1i with a repression of CDK1^Tyr15^ phosphorylation and accumulation of histone H3^Ser10^ phosphorylation. The cells eventually underwent apoptosis, as indicated by the appearance of cleaved PARP1. Although NPC cells were also more sensitive to CHK1i than nasopharyngeal epithelial cells, the differences were not as remarkable as WEE1i. The cell cycle was disrupted after NP460 cells were treated with high concentrations of CHK1i (Figure 3).

Given that NPC cells were significantly more sensitive than nasopharyngeal epithelial cells to WEE1i, we also evaluated the effects of WEE1 inhibition on NPC growth in animal tumor models. HONE1 cells were injected subcutaneously into nude mice; and WEE1i was delivered using a fractionated dose approach (administrated daily from Day = 0 to 4). Figure 4C shows that treatment with WEE1i reduced the rate of tumor growth; tumor size increased again only after the treatment ended. These results indicated that WEE1i inhibited NPC growth in xenograft mouse models.

Collectively, these data indicated as standalone agents, CHK1i and WEE1i (but not ATRi and ATMi) could stimulate mitotic catastrophe in NPC cells and inhibit NPC cell growth. Moreover, NPC cells were more sensitive to WEE1i than nasopharyngeal epithelial cells.

### Synergism in targeting CHK1/CHK2 and WEE1 together in NPC cells

**Figure 5:**
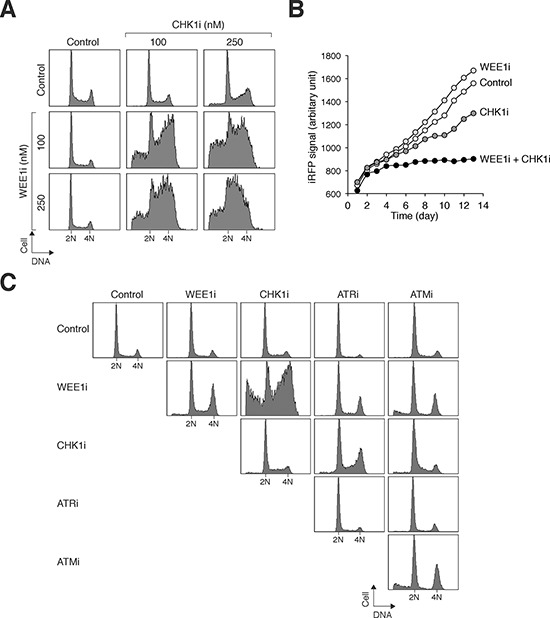
Synergism between chemicals that target CHK1/CHK2 and WEE1 in NPC cells **A.** Co-inhibition of CHK1/CHK2 and WEE1 disrupts the cell cycle. HONE1 cells were exposed to the indicated concentrations of CHK1i and WEE1i individually or in combination. After 24 h, the cells were harvested and analyzed with flow cytometry. **B.** Co-inhibition of CHK1/CHK2 and WEE1 abolishes cell proliferation. HONE1 cells expressing infrared fluorescent protein iRFP were used so that the relative cell number could be detected using infrared imaging systems. The cells (∼200) were seeded onto 6-well culture plates and cultured in the presence of the indicated combination of WEE1i (250 nM) and CHK1i (100 nM). After 24 h, the cells were washed gently and propagated in normal medium. The plate was scanned daily with an Odyssey infrared imaging system and the iRFP signal was quantified. **C.** Not all chemicals targeting the checkpoint kinase cascade show synergism. HONE1 cells were treated with combinations of WEE1i (250 nM), CHK1i (250 nM), ATRi (5 μM), and ATMi (5 μM) as indicated. The cells were harvested 24 h later for flow cytometry analysis.

A general approach for target therapies is to utilize minimum drug concentrations to reduce non-specific effects and general toxicity. A growing body of evidence indicates that targeting CHK1/CHK2 and WEE1 together can increase cytotoxicity in a variety of cancer cell types [16]. We also tested if mitotic catastrophe can be induced when CHK1/CHK2 and WEE1 are targeted together in NPC cells. Sublethal concentrations of CHK1i and WEE1i that did not trigger mitotic catastrophe on their own were used. While CHK1i or WEE1i individually did not significantly affect cell cycle progression, combination of the two inhibitors increased accumulation of cells with G_2_/M and sub-G_1_ DNA contents (Figure 5A). As anticipated from the alteration of the cell cycle, cell proliferation was reduced by combining CHK1i and WEE1i (Figure 5B). Among the checkpoint inhibitors tested, only CHK1i and WEE1i displayed significant synergism in NPC cells (Figure 5C). Collectively, these data indicate that targeting CHK1/CHK2 and WEE1 together induces more mitotic catastrophe than the individual components alone.

## DISCUSSION

Here we demonstrated that several checkpoint inhibitors could disrupt the G_2_ DNA damage checkpoint in NPC cells. These included inhibitors against WEE1, CHK1/CHK2, and ATR in both HONE1 cells (Figure 2) and HNE1 cells (Figure S2). An interesting finding is that the ATMi used (KU-60019) was not effective in disrupting the IR-induced checkpoint in NPC cells. One possibility is that both ATM and ATR were activated by IR and that ATR played a more important role in maintaining the checkpoint. In other cell types including HeLa (Figure S4) [24] and osteosarcoma [25], checkpoint abrogation by inhibition of WEE1 results in extensive mitotic catastrophe. In NPC cells, however, premature mitosis induced in the presence of DNA damage did not result in mitotic catastrophe (Figures 2, S2, S5). As relatively high dose of IR was used (10 Gy), it is unlikely that cells were able to repair the double-strand breaks at the time of checkpoint abrogation.

As a standalone agent, CHK1i is believed to trigger DNA damage through unscheduled initiation of DNA replication [15]. WEE1i has also been shown to be effective as a standalone agent in various cancer cell lines [26, 27]. Although remains to be formally established, it is likely that WEE1i also triggers DNA damage through similar mechanisms as CHK1i. It should be noted that as CHK1/CHK2 regulates both WEE1 and CDC25s, it is likely that CHK1i and WEE1i do not induce identical effects.

Several lines of evidence indicated that CHK1i and WEE1i induced mitotic catastrophe in NPC cells, including an accumulation of G_2_/M cells (Figures 3, S6), histone H3^Ser10^ phosphorylation (Figure 4A, 4B), and apoptosis (Figure 4A, 4B). Significantly, nasopharyngeal epithelial cells were less sensitive to CHK1i and WEE1i (Figure 3). The protein expression of CHK1 and WEE1 in NPC and nasopharyngeal epithelial cells (Figure 1) indicated a correlation with drug sensitivity. One possibility is that as the DNA damage checkpoint circuitry is re-wired by constitutive overexpression of checkpoint kinases, the cell may become more dependent on these kinases. Indeed, it has become increasingly clear that DNA damage checkpoints are dysregulated in NPC [17]. For example, the Epstein-Barr virus latent protein LMP1 disrupts the checkpoint by hindering CHK1 activation [28]. It has been reported that cancer cells lacking p53 are particularly sensitive to WEE1i [20]. It is noteworthy that both p53 positive (HNE1) [29] and p53-mutated (HONE1) [30] NPC cell lines were sensitive to WEE1i in this study.

Our studies also indicated that in contrast to CHK1i and WEE1i, ATRi was relatively ineffective on NPC cells (Figures 3, S6). Given that the K_i_ of the ATRi (VE-821) is 6 nM (> 600-fold selectivity over related kinases ATM or DNA-PK) [22], the concentrations used in this study were expected to be sufficient to inhibit ATR. Accordingly, the G_2_ DNA damage checkpoint was readily uncoupled by ATRi, leading to mitotic entry (Figure 2D). Although the mechanistic basis of the relatively weak cytotoxicity of ATRi compare to CHK1i/WEE1i remains to be defined, our observations suggest that targeting different components of the checkpoint kinase cascade may not be equally effective in NPC cells.

Challenging NPC cells with CHK1i and WEE1i together induced more extensive mitotic catastrophe than the individual drugs alone (Figure 5). These results are consistent with the synergistic effects of CHK1i and WEE1i observed in other cancer cell lines such as cervical carcinoma [31]. WEE1i (MK-1775) also acts synergistically with other CHK1 inhibitors including AR458323 [32], PF-00477736 [33] [34], and MK-8776 [35] in reducing cell growth in a variety of cancers. Our results suggest that although NPC cells already appeared to be more sensitive to WEE1i than non-cancer cells, the cytotoxicity could be further increased by combinatorial treatment with CHK1i.

## MATERIALS AND METHODS

### Cell culture

NPC cell lines C666-1 [36], CNE2 [37], HNE1 [19], and HONE1 [19] were obtained from NPC AoE Cell Line Repository (The University of Hong Kong). The HeLa used in this study was a clone that expressed the tTA tetracycline repressor chimera [38]. Normal human lymphoblastoid cells (GM03798), and A-T lymphoblastoid cells (GM03189D) were obtained from Coriell Cell Repositories (Camden, NJ, USA). Cells were propagated in RPMI1640 (for C666-1 and lymphoblasts) or DMEM (for other cell lines) supplemented with 10%(v/v) calf serum (Life Technologies, Carlsbad, CA, USA) (for HeLa) or 5%(v/v) calf serum and 5%(v/v) fetal bovine serum (Life Technologies) (for CNE2 and HNE1), 15%(v/v) fetal bovine serum (for lymphoblasts), or 10%(v/v) fetal bovine serum (for other cell lines) and 50 U/ml penicillin streptomycin (Life Technologies). Telomerase-immortalized nasopharyngeal epithelial cell lines NP361, NP460, and NP550 [39] were propagated in keratinocyte serum-free medium supplemented with 50% v/v Epilife and 50 U/ml penicillin-streptomycin (Life Technologies).

HeLa cells stably expressing histone H2B-GFP were generated as described previously [40]. HONE1 and HNE1 expressing histone H2B-mRFP were generated by infecting the cells with histone H2B-mRFP-expressing retroviruses in the presence of 5 μg/ml of polybrene (Sigma-Aldrich). The transduced cells were selected with 200 μg/ml of hygromycin B (Life Technologies) for ∼2 weeks before individual colonies were isolated. A NP460 cell line expressing histone H2B-GFP was generated by infecting NP460 cells with histone H2B-GFP-expressing retroviruses in the presence of 5 μg/ml of polybrene. The transduced cells were selected with 5 μg/ml of blasticidin (Life Technologies) for ∼2 weeks before individual colonies were isolated. HONE1 cells expressing iRFP were generated by transfecting cells with an iRFP-expressing construct [41] followed by sorting with a flow cytometer (FACSAria IIIu, Becton Dickinson, Franklin Lakes, NJ, USA) using a 633-nm laser for excitation. The cells were propagated for one week before being sorted again. Three rounds of sorting were performed.

Unless stated otherwise, cells were treated with the following reagents at the indicated final concentration: AZD7762 (Selleck Chemicals Houston, TX, USA), KU-60019 (Selleck Chemicals; 5 μM), MK-1775 (Selleck Chemicals), nocodazole (Sigma-Aldrich; 0.1 μg/ml), and VE-821 (Selleck Chemicals; 2.5 μM). Trypan blue analysis [42] and preparation of cell-free extracts were performed as described previously [43].

### RNA interference

Cells were transfected with siRNA (10 nM) using Lipofectamine™ RNAiMAX (Life Technologies, Carlsbad, CA, USA). Stealth siRNA targeting CHK1 (GGCUUGGCAACAGUAUUUCGGUAUA) and WEE1 (CCUCAGGACAGUGUCGUCGUAGAAA) were obtained from Life Technologies.

### Flow cytometry

Flow cytometry analysis after propidium iodide staining was performed as described previously [42].

### Ionizing radiation

IR was delivered with a caesium^137^ source from a MDS Nordion (Ottawa, Canada) Gammacell 1000 Elite Irradiator.

### Infrared imaging

Cell proliferation was measured using a method involving the expression of an infrared fluorescent protein iRFP in cells [41, 44]. Infrared signals were quantified with an Odyssey CLx system (LI-COR Biosciences, Lincoln, NE, USA).

### Live-cell imaging

Cells were seeded onto 24-well culture plates and imaged using a Ti-E inverted fluorescence microscope (Nikon, Tokyo, Japan) equipped with an ultra-low noise sCMOS camera (Andor Technology, Belfast, UK) and a TC temperature, humidity, and CO_2_ control system (Chamlide, Live Cell Instrument, Seoul, Korea). Data acquisition was carried out at 5 min/frame.

### Antibodies and immunological methods

Antibodies against CDK1 [45] and cyclin B1 [40] were obtained from sources as described previously. Antibodies against β-actin (Sigma-Aldrich), γH2AX (Bethyl Laboratories, Montgomery, TX, USA), ATR, ATM, CHK1, CHK2, phospho-histone H3^Ser10^, and WEE1 (Santa Cruz Biotechnology, Santa Cruz, CA, USA), phospho-CDK1^Tyr15^ and cleaved PARP1(Asp214) (BD Biosciences, Franklin Lakes, NJ, USA) were obtained from the indicated suppliers. Immunoblotting was performed as described [43].

### Tumor xenograft

HONE1 cells (2 × 10^7^) were injected subcutaneously into both sides of the dorsa of 4–6-week-old female BALB/c athymic (nude) mice. Tumors were measured using a Vernier caliper. Volume was calculated according to the formula: π/6 × length × width^2^ [46]. Day = 0 was designated as when the tumor volume was ∼100 mm^3^. MK-1775 (50 mg/kg, i.p.) was administrated daily from Day = 0 to 4. Mice were killed when tumors in the control group reached 1,000 mm^3^.

## SUPPLEMENTARY FIGURES


