# Supplementary material for: Pharmacological inactivation of CHK1 and WEE1 induces mitotic catastrophe in nasopharyngeal carcinoma cells
Source: Oncotarget. 2015 May 21;6(25):21074–84. doi: 10.18632/oncotarget.4020 (PMC4673251; doi:10.18632/oncotarget.4020)
Supplement: Supplementary file 1 [file oncotarget-06-21074-s001.pdf]

SUPPLEMENTARY FIGURES

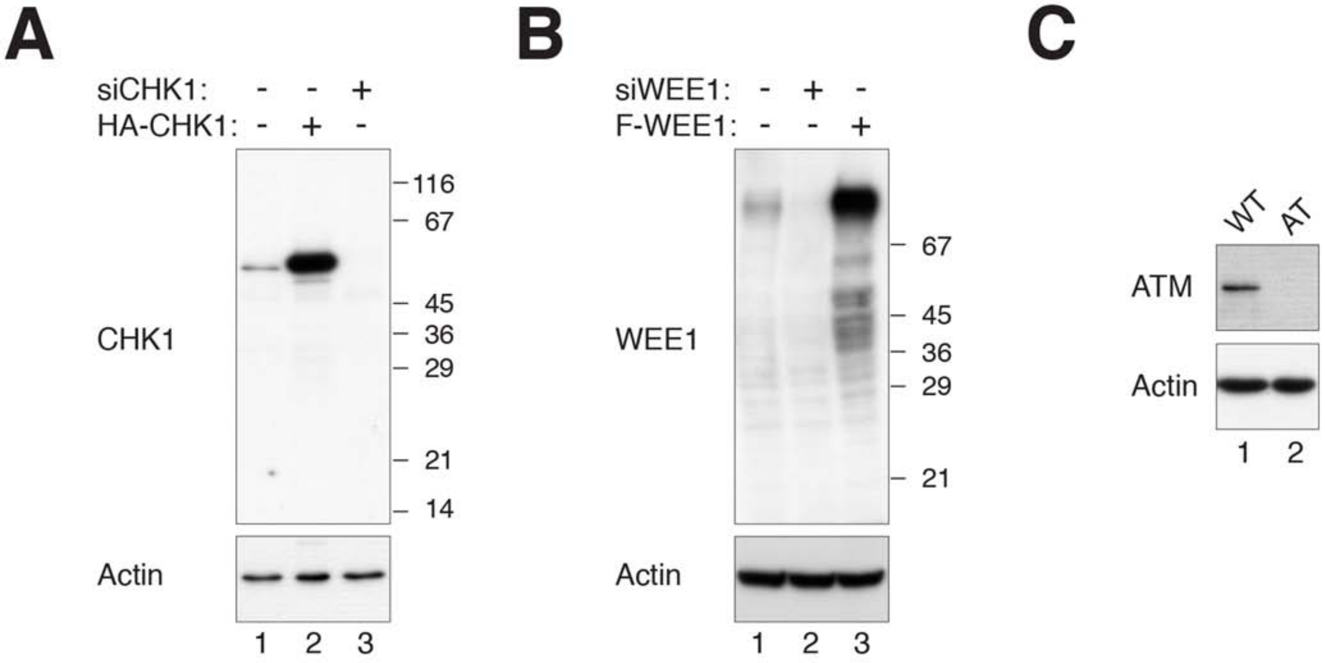

Supplementary Figure S1: Specificity of the CHK1 and WEE1 antibodies. A. Specificity of CHK1 antibodies. HeLa cells were transfected with either a plasmid expressing HA-CHK1 or siRNA against CHK1 (siCHK1). Lysates were prepared and analyzed with immunoblotting using antibodies against CHK1. Actin analysis was included to assess protein loading and transfer. The positions of molecular size standards (in kDa) are indicated. **B.** Specificity of WEE1 antibodies. HeLa cells were transfected with either a plasmid expressing FLAG-WEE1 (F-WEE1) or siRNA against WEE1 (siWEE1). Lysates were prepared and analyzed with immunoblotting using antibodies against WEE1. Actin analysis was included to assess protein loading and transfer. The positions of molecular size standards (in kDa) are indicated. **C.** Specificity of ATM antibodies. Lysates from normal human lymphoblastoid cells (WT) and A-T lymphoblastoid cells (AT) were analyzed with immunoblotting using antibodies against ATM. Actin analysis was included to assess protein loading and transfer.

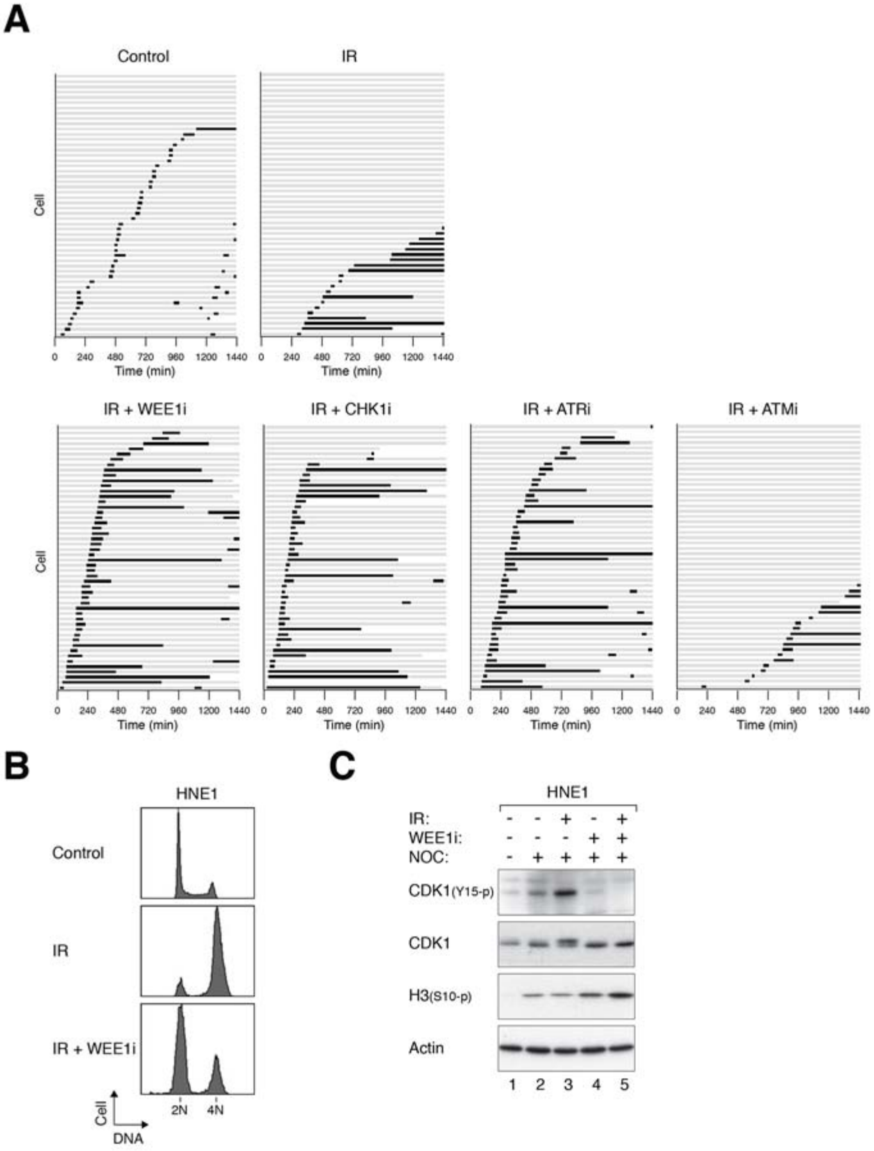

Supplementary Figure S2: Targeting ATR, CHK1/CHK2, and WEE1 abrogates the G_2 DNA damage checkpoint in irradiated HNE1 cells. **A.** HNE1 cells expressing histone H2B-GFP were either mock-treated or irradiated with 10 Gy of IR. After 16 h, the cells were incubated with buffer, WEE1i (250 nM), CHK1i (250 nM), ATRi (5 μM), or ATM (5 μM). Individual cells were then tracked for 24 h with time-lapse microscopy. Each horizontal bar represents one cell ($n = 50$). Light grey: interphase; black: mitosis (from DNA condensation to anaphase); truncated bars: cell death. Note that only one of the daughter cells was tracked after the first mitosis.**B.** Inhibition of WEE1 overcomes IR-induced G_2 arrest. HNE1 cells were either mock-treated or irradiated with 10 Gy of IR. After 16 h, the cells were incubated with buffer or WEE1i (250 nM). After 6 h, the cells were harvested and analyzed with flow cytometry. **C.** Disruption of the G_2 DNA damage checkpoint by inhibition of WEE1. HNE1 cells were either mock-treated or irradiated with 10 Gy of IR. After 16 h, the cells were incubated with either buffer or 250 nM of WEE1i. Nocodazole was also applied to trap cells in mitosis. The cells were harvested after another 6 h. Lysates were prepared and the indicated proteins were detected with immunoblotting. Uniform loading of lysates was confirmed by immunoblotting for actin.

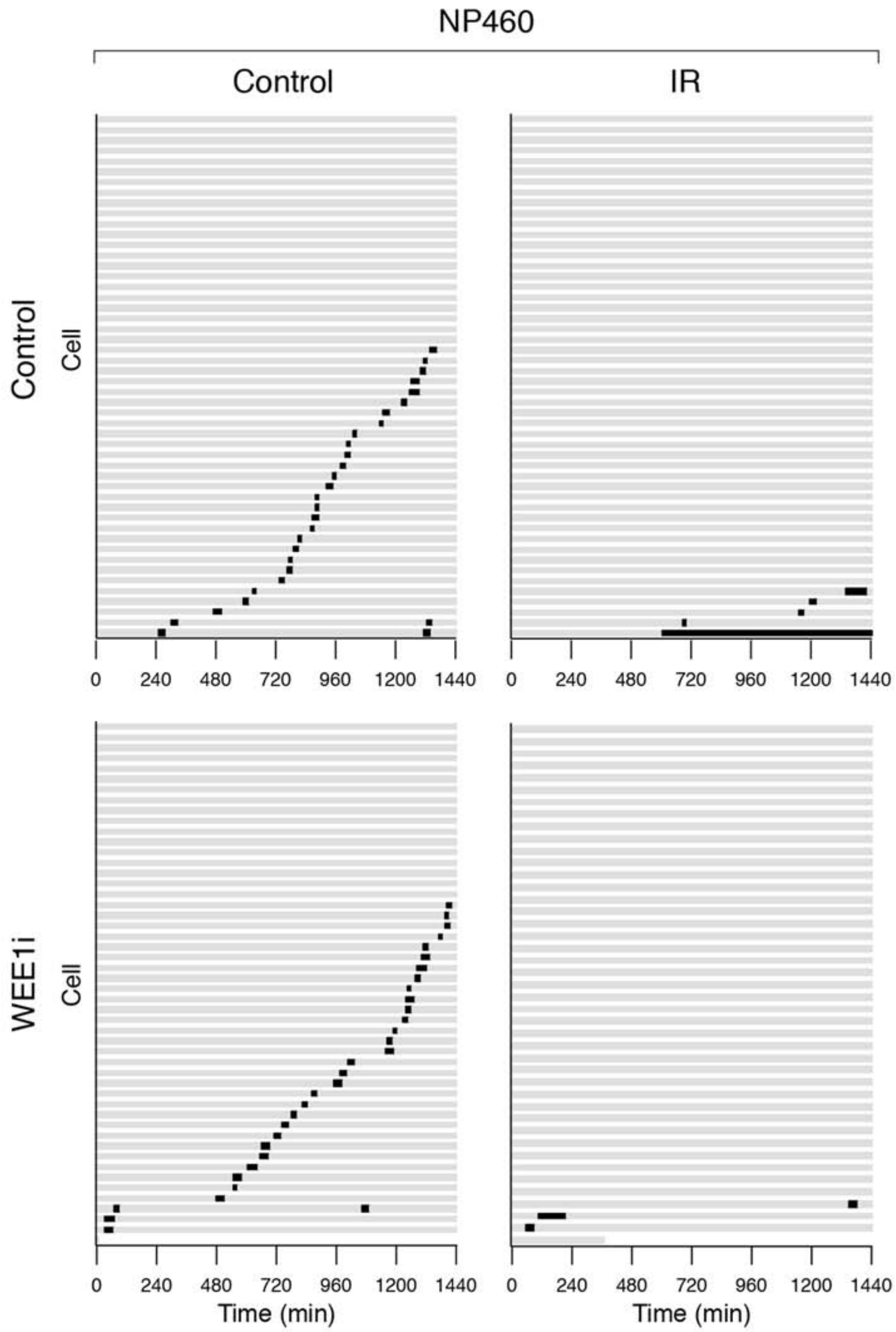

Supplementary Figure S3: WEE1i does not bypass the IR-mediated G_2 arrest in NP460 cells. NP460 cells expressing histone H2B-GFP were either mock-treated or irradiated with 10 Gy of IR. After 16 h, the cells were incubated with either buffer or WEE1i (100 nM). Individual cells were then tracked for 24 h with time-lapse microscopy. Each horizontal bar represents one cell ($n = 40 - 50$). Light grey: interphase; black: mitosis (from DNA condensation to anaphase); truncated bars: cell death. Note that only one of the daughter cells was tracked after mitosis.

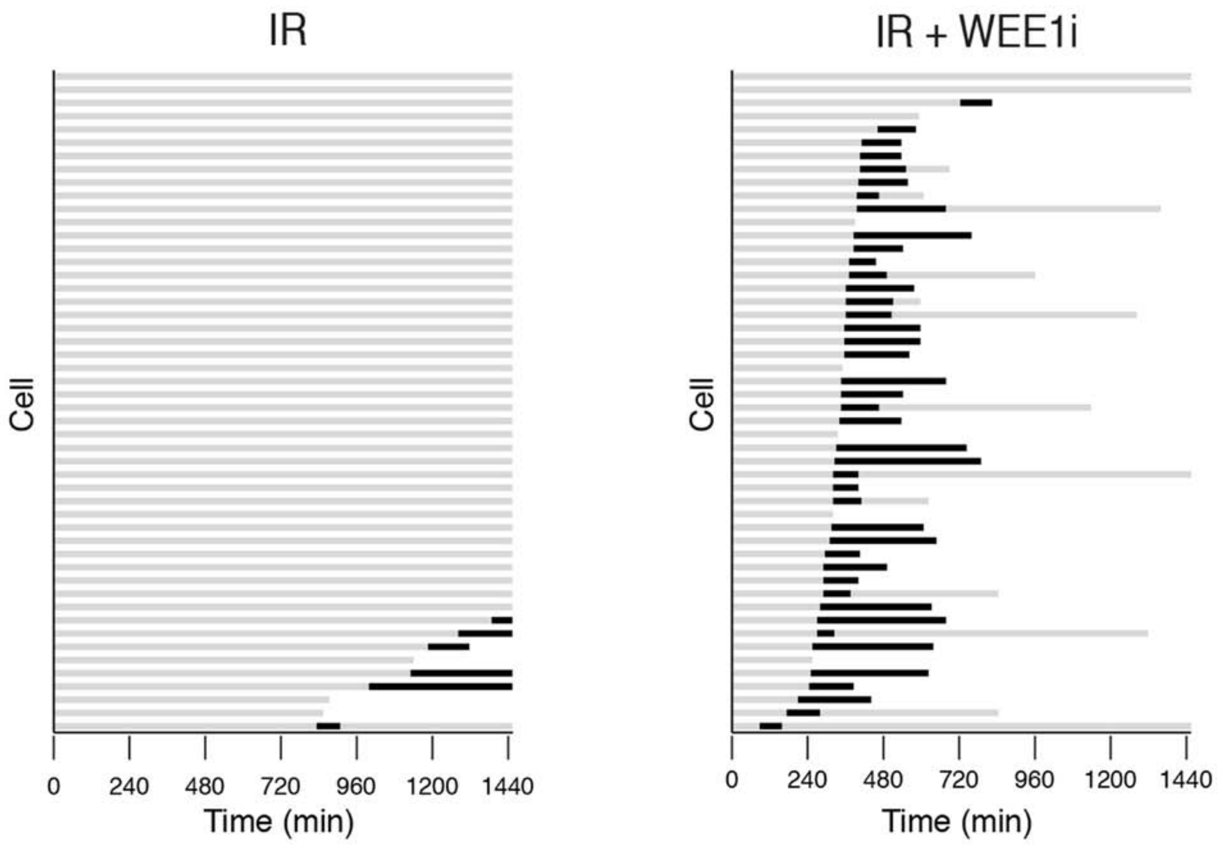

Supplementary Figure S4: Abrogation of the G_2 DNA damage checkpoint induces massive mitotic catastrophe in HeLa cells. HeLa cells expressing histone H2B-GFP were irradiated with 15 Gy of IR. After 16 h, the cells were incubated with either buffer or WEE1i (100 nM). Individual cells were then tracked for 24 h with time-lapse microscopy. Each horizontal bar represents one cell (n = 50). Light grey: interphase; black: mitosis (from DNA condensation to anaphase); truncated bars: cell death. Note that only one of the daughter cells was tracked after mitosis.

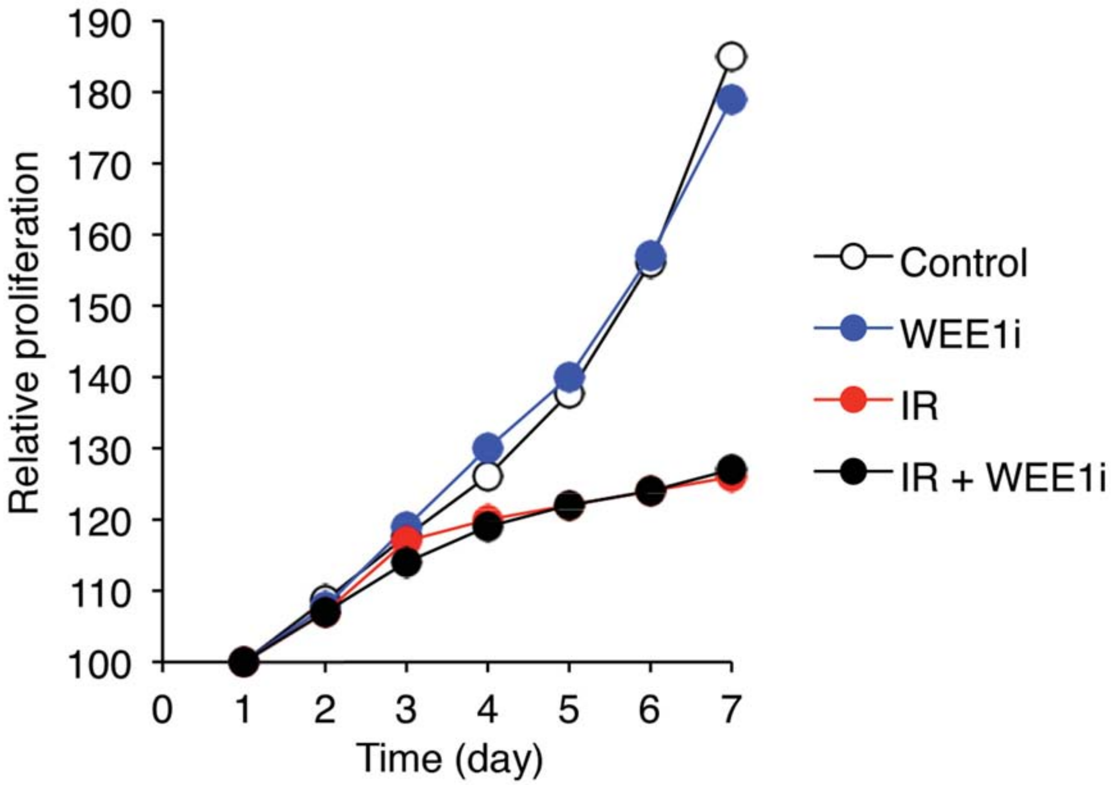

Supplementary Figure S5: Abrogation of the G_2 DNA damage checkpoint does not reduce viability in HONE1 cells. HONE1 cells expressing iRFP were either mock-treated or irradiated with 10 Gy of IR and incubated with either buffer or 100 nM of WEE1i. Over the next six days, the plate was scanned daily with an Odyssey infrared imaging system and the iRFP signal was quantified (normalized to the signal at day 1).

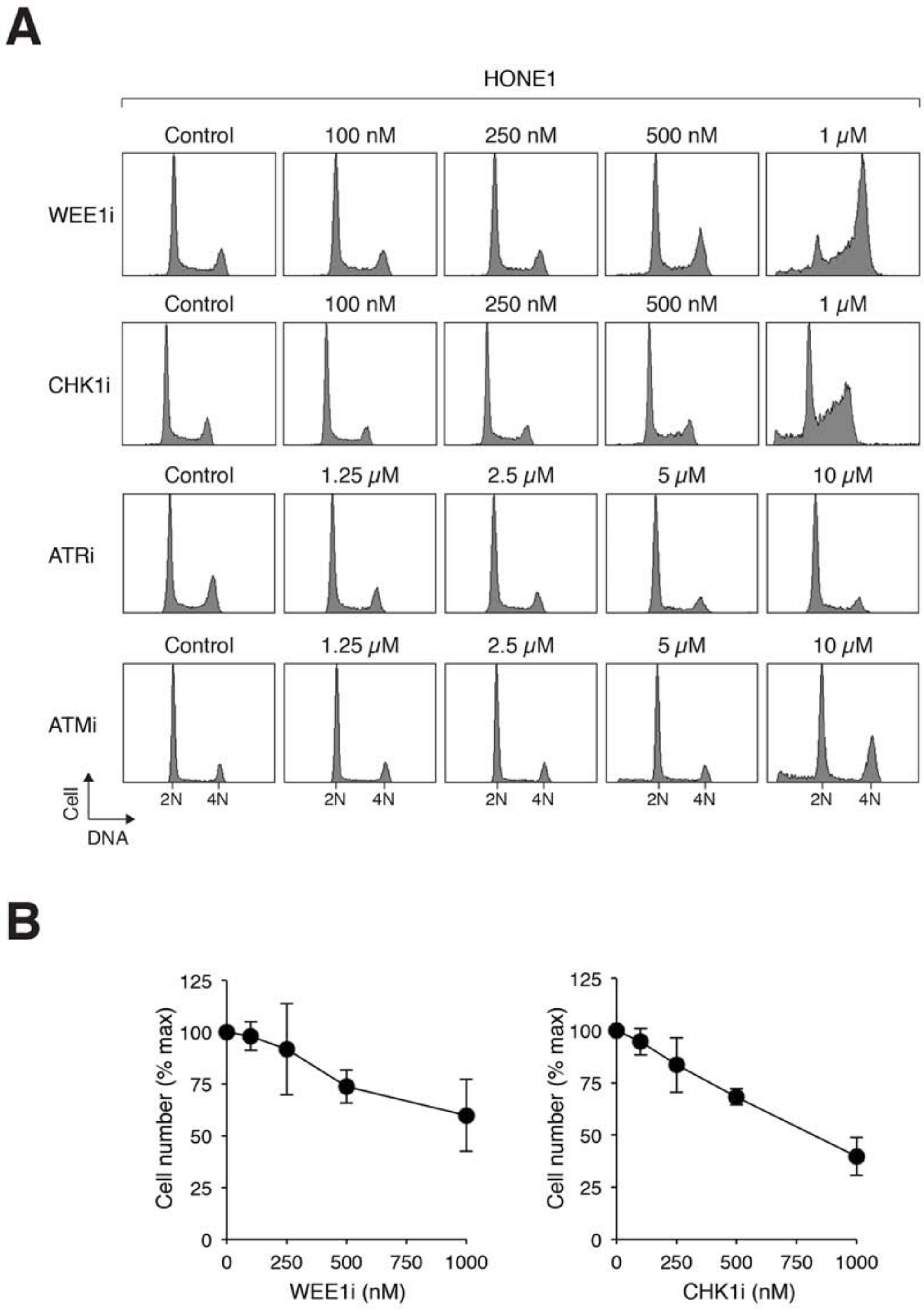

Supplementary Figure S6: Inhibition of CHK1/CHK2 and WEE1 sensitizes HONE1 cells. A. HONE1 cells were exposed to the indicated concentrations of MK-1775 (WEE1i), AZD7762 (CHK1i), VE-821 (ATRi), or KU-60019 (ATMi). After 24 h, the cells were harvested and analyzed with flow cytometry. The positions of 2N and 4N DNA contents are indicated. **B.** HONE1 cells were exposed to different concentrations of MK-1775 (WEE1i) or AZD7762 (CHK1i). After 24 h, the number of viable cells was analyzed with trypan blue staining and cell counting. Mean ± SD of three independent experiments.
